# Meteorological Factors Influence the Presence of Fungi in the Air; A 14-Month Surveillance Study at an Adult Cystic Fibrosis Center

**DOI:** 10.3389/fcimb.2021.759944

**Published:** 2021-11-26

**Authors:** Norman van Rhijn, James Coleman, Lisa Collier, Caroline Moore, Malcolm D. Richardson, Rowland J. Bright-Thomas, Andrew M. Jones

**Affiliations:** ^1^ Manchester Fungal Infection Group, Division of Infection, Immunity and Respiratory Medicine, University of Manchester, Manchester, United Kingdom; ^2^ Manchester Adult Cystic Fibrosis Centre, Manchester University National Health Service (NHS) Foundation Trust, Manchester, United Kingdom; ^3^ Faculty of Biology Medicine and Health, The University of Manchester, Manchester, United Kingdom; ^4^ Mycology Reference Centre, European Confederation of Medical Mycology (ECMM) Excellence Centre of Medical Mycology, Manchester University National Health Service (NHS) Foundation Trust, Manchester, United Kingdom

**Keywords:** *Aspergillus fumigatus*, *Penicillium*, fungi, air sampling, temperature, cystic fibrosis, climate, weather

## Abstract

**Background:**

Cystic fibrosis is an inherited disease that predisposes to progressive lung damage. Cystic fibrosis patients are particularly prone to developing pulmonary infections. Fungal species are commonly isolated in lower airway samples from patients with cystic fibrosis. Fungal spores are prevalent in the air.

**Methods:**

We performed environmental air sampling surveillance at the Manchester Adult Cystic Fibrosis Centre, UK (MACFC) over a 14-month period to assess fungal growth inside and outside the CF center.

**Results:**

Airborne counts of fungal spores peaked from May to October, both in outdoor and indoor samples. Collection of meteorological data allowed us to correlate fungal presence in the air with elevated temperatures and low wind speeds. Additionally, we demonstrated patient rooms containing windows had elevated fungal counts compared to rooms not directly connected to the outdoors.

**Conclusions:**

This study suggests that airborne *Aspergillus fumigatus* spores were more abundant during the summer months of the survey period, which appeared to be driven by increased temperatures and lower wind speeds. Indoor counts directly correlated to outdoor *A. fumigatus* levels and were elevated in patient rooms that were directly connected to the outdoor environment *via* an openable window designed for ventilation purposes. Further studies are required to determine the clinical implications of these findings for cystic fibrosis patients who are predisposed to *Aspergillus* related diseases, and in particular whether there is seasonal influence on incidence of *Aspergillus* related conditions and if screening for such complications such be increased during summer months and precautions intensified for those with a known history of *Aspergillus* related disease.

## Introduction

Cystic Fibrosis (CF) is a life-long inherited disorder affecting over 10,000 people in the United Kingdom and more than 70,000 people worldwide (Bobadilla et al., 2002; Taylor-Robinson et al., 2018). CF is caused by a mutation in the gene encoding the cystic fibrosis transmembrane conductance regulator (CFTR) protein (Kerem et al., 1989; Riordan et al., 1989). The CFTR protein is involved in intracellular calcium homeostasis and acts as a cyclic adenosine monophosphate-dependent ion channel, controlling the transport of salts and water across epithelial cell membranes (Grubb and Boucher, 1999). Mutations in the protein leads to defective ion flux, resulting in thickened mucus and impaired mucociliary clearance of particles and pathogens. Patients with CF are predisposed to recurrent and chronic infections which, together with an exaggerated host inflammatory response leads to progressive airway damage and eventually respiratory failure (Cohen and Prince, 2012; Taylor-Robinson et al., 2018).

In recent years, the life expectancy of CF patients has increased dramatically, with a median predicted survival of 49 years (McCormick et al., 2002; Dodge et al., 2007; Taylor-Robinson et al., 2018). Approximately 90% of CF mortality is attributed to respiratory failure secondary to chronic or recurrent infections (Cantin et al., 2015; Beswick et al., 2020). *Aspergillus fumigatus* is commonly isolated from lower respiratory tract samples (Bakare et al., 2003; Armstead et al., 2014) and can cause a range of diseases within CF patients, mainly but not exclusively: allergic bronchopulmonary aspergillosis (ABPA), *Aspergillus* bronchitis, and sensitization (Baxter et al., 2013; King et al., 2016). ABPA results from hypersensitivity to *Aspergillus* spp., occurring in 6–25% of CF patients, most of whom are adolescents or adults (de Almeida et al., 2006; Maturu and Agarwal, 2015). *Aspergillus* bronchitis affects approximately 9% of CF patients, while sensitization can be found in approximately 39% of CF patients (Maturu and Agarwal, 2015; Brandt et al., 2018). However, there is a large variation of reported prevalence of *Aspergillus* infection in CF in the literature, probably due to variable methodologies both for detecting the presence of and host reaction to *Aspergillus* and furthermore inconsistent diagnostic criteria (Mastella et al., 2000; Stevens et al., 2003; Patterson and Strek, 2010).

Risk factors for isolation of *Aspergillus* in respiratory samples in CF patients have been attributed to inhaled antibiotics, oral corticosteroid treatment, and exocrine pancreatic insufficiency (Brenier-Pinchart et al., 2009; Hong et al., 2018). Meteorological parameters have been reported to influence fungal contamination in other healthcare settings (van Rhijn and Bromley, 2021) but not in CF setting. Higher outdoor mean and maximum temperatures, and also indoor temperatures, are associated with an increased fungal presence (Li and Kendrick, 1995; Singh and Chauhan, 2013; Alshareef and Robson, 2014). In several studies, humidity and rainfall have been correlated to higher fungal load in the atmosphere (Li and Kendrick, 1995; Takahashi, 1997; Brenier-Pinchart et al., 2009). Climate change may also be changing our daily exposure to fungi (van Rhijn and Bromley, 2021). Construction and demolition work have been associated with an increased isolation of fungi in air samples (Srinivasan et al., 2002; Curtis et al., 2005; Pilmis et al., 2017; Wirmann et al., 2018). Building works adjacent to healthcare settings are an important risk factor for nosocomial aspergillosis. Preventative measures, such as laminar airflow and HEPA filters, have been explored to attempt to keep indoor fungal loads consistently low during construction work (Streifel et al., 1989; Cornet et al., 1999; Falvey and Streifel, 2007). In this study, we examined the relationship between meteorological factors with environmental load of *A. fumigatus* and other fungi at the MACFC over a 14-month period.

## Materials and Methods

### Air Sampling

**Figure 1:**
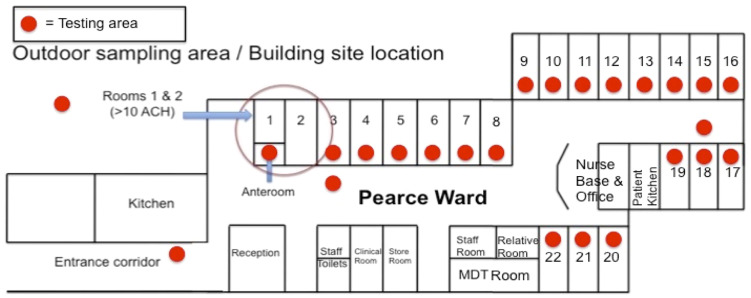
Layout of the Pearce Ward at MACFC. A schematic representation of the Pearce Ward at MACFC. Red dots represent the sampling locations used for this study. Orange bars represent windows. The anteroom into Rooms 1 and 2 has 10 air changes per hour (ACH), compared to 1.7 ACH for other rooms.

All sampling took place in the adult CF ward at the Wythenshawe Hospital, Manchester University Hospitals NHS Foundation Trust, which houses both the Manchester Adult Cystic Fibrosis Centre and the National Aspergillosis Centre. The hospital is situated in a mixed residential and rural area of south Manchester. The CF center comprises of a 22-bedded CF inpatient ward on the ground level and outpatient department and offices, situated directly above the ward on the first floor. There is a specific car park for CF patients, located opposite the ward entrance, to prevent patients from having to walk through the main hospital. Sampling was performed in the CF ward at regular intervals at consistent sites: 18 out of 22 inpatient rooms, two sites in the ward corridor, inside an anteroom that provides positive pressure (>10 air changes per hour) to two inpatient rooms, and outside the kitchen on the ward (**Figure 1**). Samples were also taken in the CF patients’ car park. Air samples were taken using a single headed SAS microbial air sampler (PBI, Milan, Italy), which used impaction onto a malt agar plate (Cherwell labs) and samples 1 m^3^ air over 10 min. Plates were incubated at 30°C for 4 days and sampled at a height of 1.2–1.5 m (Morris et al., 2000).

Fungal species were determined by macroscopic phenotypic examination and microscopic examination at the Mycology Reference Centre. Lactophenol blue was added to visualize fungal elements. Colony counts are expressed as colony forming units (CFU).

### Meteorological Data

Meteorological data was obtained from the Met Office (Met Office, United Kingdom) from the Rostherne No. 2 (Cheshire East) weather station (Lat: 53.336, Long: −2.3833), which is located approximately 6.6 km/4.10 miles away in a straight line from the Wythenshawe Hospital site. In total, 10 different meteorological variables were used: daily maximum, minimum, and mean temperatures (09:00–09:00) in Celsius; daily total rainfall (09:00–09:00) in mm; daily total sunshine (01:00–24:00) in hours; daily mean wind speed and maximum gust (01:00–24:00) in knots (kn); and daily mean, maximum and minimum relative humidity (00:00–23:00) in %. For each variable, values were used for both each sampling date as well as the day before each sample was undertaken.

### Data Analysis

Data was analyzed using R and Rstudio, using packages ggplot2, tidyverse, and corrplot (Wickham, 2011; Wei et al., 2017; Wickham et al., 2019). Correlation analysis was performed using multiple non-linear regression, with p <0.05 being deemed statistically significant. The growing season was defined as May to October to subset the data. For the analysis of correlation of meteorological parameters to indoor CFUs, data from the anteroom was excluded. Data is presented as mean (SD) unless stated otherwise.

## Results

### Outdoor Fungal Spores Correlate With Elevated Temperature and Low Wind Speeds

**Figure 2:**
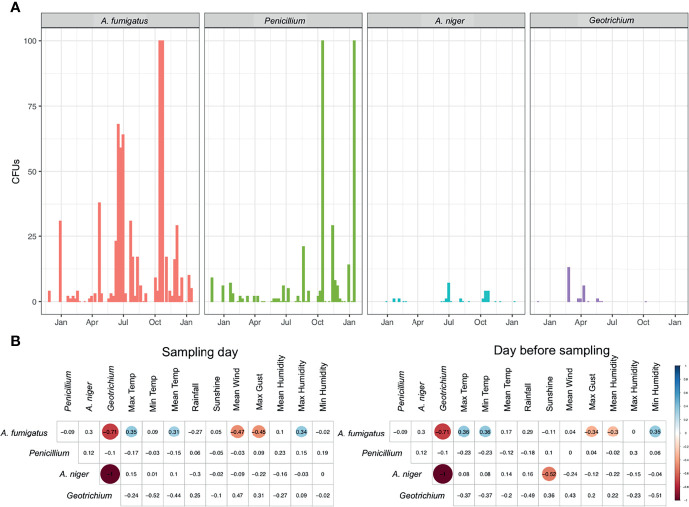
Presence of fungal spores in outdoor air. **(A)** CFUs of *A. fumigatus, Penicillium*, *A. niger*, and *Geotrichium* in outdoor air samples measured over a 14-month period. **(B)** Spearman’s rank correlation of CFUs from fungi in outdoor air samples to meteorological parameters collected on the day of sampling or the day before sampling. Statistically significant (P < 0.05) correlations are shown in circles, with blue showing positive correlations and red negative correlations. Correlation values are shown and the size of the circle corresponds to this value.

Environmental surveillance to quantify the abundance of fungal spores in the air was set up in and around the MACFC during November 2014–January 2016. Sampling occurred on a total of 48 dates from November 27, 2014 until January 15, 2016, with samples mostly being taken weekly (mean gap between samples 8.8 ± 4.55 days, median: 7 days (range 6–28 days), with allowances for holidays such as Christmas and Easter (**Figure 1** and **Supplemental Data 1**). Of all the culturable fungi, the most abundant fungal species detected was *A. fumigatus* [mean (SD) 16.23 (25.14) colony forming unites (CFUs)], followed by *Penicillium* species (8.47 (32.42) CFUs) and *Geotrichum* (2.2 (3.99) CFUs). Peak spore counts of *Aspergillus* species occurred during the summer and autumn months (end of May–October), while spores of *Penicillium* species peaked during autumn and winter (October to early January). *Geotrichum* was detected more during spring and negatively correlated with the presence of *A. fumigatus* (R = −0.71, p < 0.05) and *Aspergillus niger* in the air (R = −1, p <0.05) (**Figure 2A**).

Throughout the year, we were able to positively correlate *A. fumigatus* spore abundance with maximum humidity (R = 0.34), maximum temperature (R = 0.35), and mean temperature (R = 0.31) during the sampling day (**Figure 2B** and **Supplemental Figure 1**). Spore abundance was negatively correlated with maximum gust (R = −0.45) and mean wind speed (R = −0.47) on the day of sampling. On the day prior to sampling, a positive correlation was found for *A. fumigatus* spore abundance with maximum temperature (R = 0.36), minimum humidity (R = 0.35), and minimum temperature (R = 0.36). A negative correlation was found for maximum gust (R = −0.34) and mean humidity (R = −0.3). A negative correlation for maximum gust and positive correlation for maximum temperature was found for both sampling day and the day before sampling. For *A. niger* we were able to negatively correlate sunshine on the day before sampling with abundance in air samples (R = −0.52) (**Figure 2B**).

**Figure 3:**
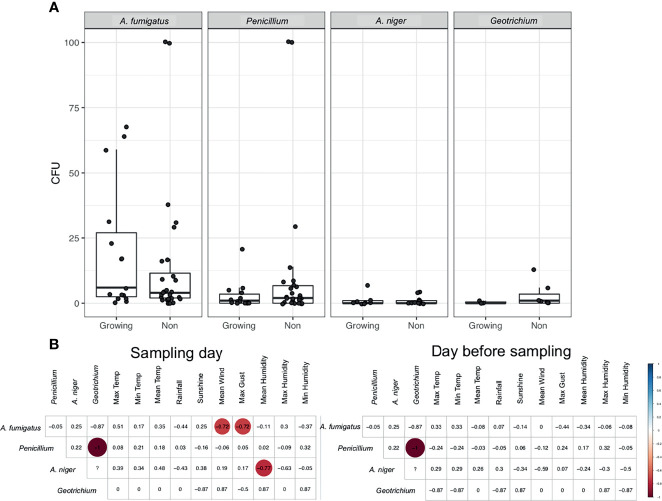
Fungal spores during the growing season. **(A)** CFUs of selected fungi during the growing season and outside of the growing season. **(B)** Spearman’s rank correlation of CFUs from fungi during the growing season to meteorological parameters collected on the day of sampling or the day before sampling. Statistically significant (P < 0.05) correlations are shown in circles, with blue showing positive correlations and red negative correlations. Correlation values are shown and the size of the circle corresponds to this value.

Temperature was clearly an important factor for *A. fumigatus* abundance and we found a seasonal pattern for all fungi observed. Therefore, we assessed the difference in fungal abundance in air during the growing season (May–October) and non-growing season (October–April). An increase in *A. fumigatus* CFUs during the growing season (mean (SD) 19.93 (23.64) CFUs), compared to non-growing season (mean (SD) 14.25 (25.70) CFUs) was observed (**Figure 3A**). For other fungal species this trend was not observed, with consistent CFUs in both growing and non-growing season. *A. fumigatus* CFUs negatively correlated with the maximum gust (R = −0.72) and mean speed of the wind (R = −0.72) during the growing season, no other significant correlation could be found for *A. fumigatus* (**Figure 3B** and **Supplemental Figure 2**). However, no significant differences were observed for differences in wind direction (**Supplemental Figure 3**).

### The Presence of *A. fumigatus* Spores in Indoor Air is Determined by Meteorological Factors and the Presence of Windows

**Figure 4:**
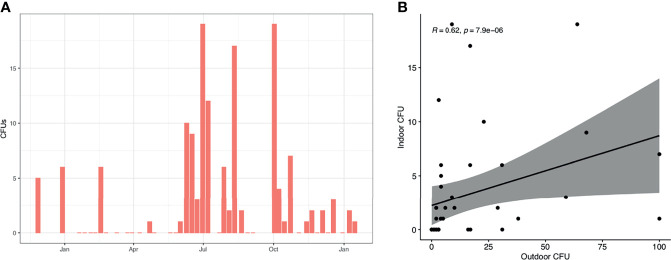
*A. fumigatus* spores in indoor air samples. **(A)** CFUs of *A. fumigatus* detected in indoor air samples. Shown is the median of all indoor samples on each date. **(B)** The presence of *A. fumigatus* in indoor and outdoor samples, matched by sampling date. Spearman’s rank correlation is shown in top left corner.

Indoor counts of *A. fumigatus* were generally lower compared to outdoor counts (mean (SD) 3.21 (5.04) *vs* 16.23 (25.15) CFUs, p < 0.0001 Wilcoxon matched-pairs signed rank test) (**Figure 4A**). Indoor CFUs of *A. fumigatus* peaked from May to November in line with outdoor counts. Indoor and outdoor *A. fumigatus* correlated when matched for their sampling date (R = 0.62, p = 7.9e−06, Spearman Rank) (**Figure 4B**).

**Figure 5:**
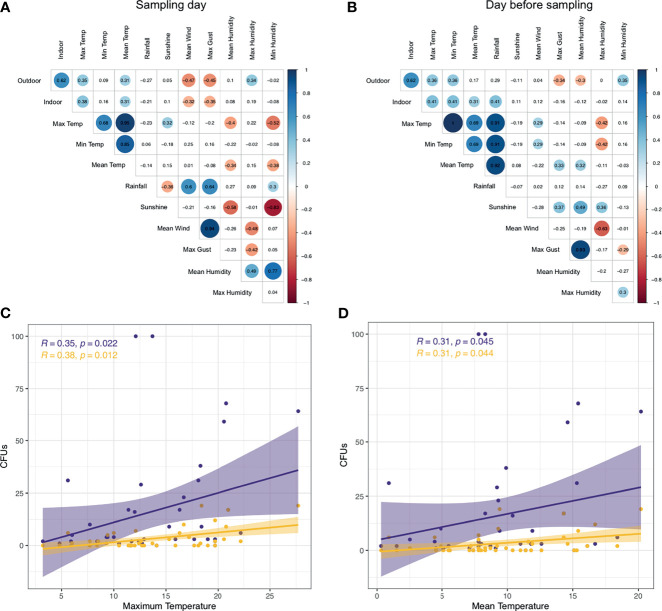
*A. fumigatus* abundance correlates with temperature and wind. **(A, B)** Correlation matrix *A. fumigatus* outdoor and indoor CFUs to meteorological parameters on the day of sampling **(A)** or day before sampling **(B)**. Statistically significant (P < 0.05) correlations by Spearman’s rank correlation are shown in circles, with blue showing positive correlations and red negative correlations. Correlation values are shown and the size of the circle corresponds to this value. **(C, D)** CFUs of *A. fumigatus* in indoor (yellow) and outdoor (purple) air samples correlate to maximum temperature **(C)** and mean temperature **(D)**. Shown in the top left are correlation values and P-value by Spearman’s rank correlation.

Examining indoor *A. fumigatus* CFUs with meteorological parameters of the sampling day and day before sampling we identified indoor CFUs correlated positively to maximum (R = 0.38) and mean temperature (R = 0.31) similar to outdoor counts. Also, a negative correlation was found for maximum gust (R = −0.35) and mean wind speed (R = −0.32), in line with our findings for outdoor CFUs (**Figure 5A**). Maximum and mean temperature correlate with each other (R = 0.95) as well as maximum gust and mean wind speed (R = 0.94) (**Figure 5B**). *A. fumigatus* CFUs the day before sampling positively correlated with maximum (R = 0.41) and mean temperature (R = 0.31), like the day of sampling, but also with the minimum temperature (R = 0.41) and rainfall (R = 0.41). Rainfall correlated strongly with the maximum (R = 0.91), minimum (R = 0.91), and mean temperature (R = 0.92).

We further explored the correlation between *A. fumigatus* CFUs indoors, outdoors and maximum or mean temperature. Increased maximum temperature correlated with elevated *A. fumigatus* CFUs in outdoor and indoor air (**Figure 5C**). Similarly, higher mean temperatures resulted in higher *A. fumigatus* CFUs indoors and outdoors with a similar rate (R = 0.31 *vs* R = 0.31) (**Figure 5D**). However, indoor sampling resulted in 18 samples with no *A. fumigatus* CFUs detected, unlike outdoors where we could detect *A. fumigatus* in more than 95% of sampling dates (no CFUs in four samples).

**Figure 6:**
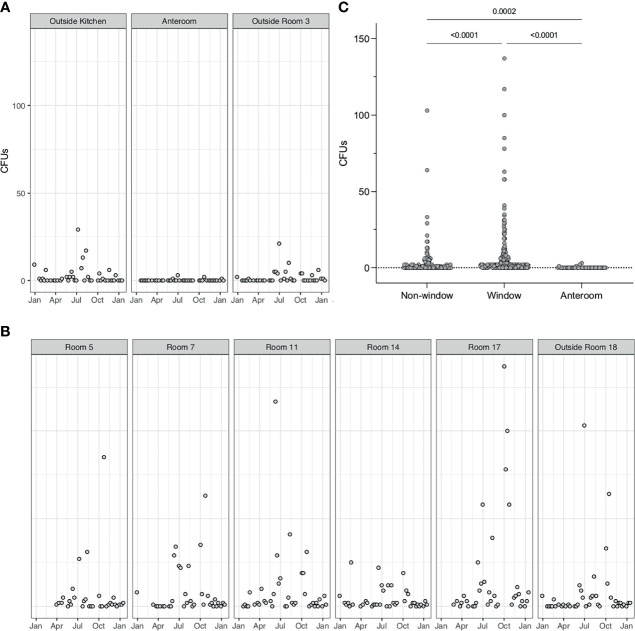
*A. fumigatus* spores are elevated in rooms with windows. **(A)**. *A. fumigatus* CFUs from air samples or rooms not directly adjacent to windows (Outside room 3 and Outside kitchen) or with positive pressure (Anteroom). Positive pressure results in close to 0 CFUs in air samples. **(B)**. *A. fumigatus* CFUs in air samples from patients’ rooms containing windows. **(C)** Comparison of *A. fumigatus* CFUs in rooms with windows and without windows, tested by Mann–Whitney-U test (P < 0.0001).

Three sampling locations were not in rooms that were not directly connected to the outside, and also an anteroom with positive pressure applied, were assessed for *A. fumigatus* CFUs. In these rooms, small numbers of CFUs (maximum detected 28 CFUs) could be detected over the course of 9 months (**Figure 6A**). In patient rooms that were directly connected to the outside *via* openable windows showed significantly higher CFUs for *A. fumigatus*, especially during the summer months (p < 0.0001) (**Figure 6B**). Even though all these rooms were considered identical, we detected variability of *A. fumigatus* CFUs between rooms. For example, in one patient bedroom (room 14) much lower counts were observed (max count = 25), than another patient bedroom where (room 17) up to 150 CFUs could be detected. A direct comparison of *A. fumigatus* CFUs between rooms that contained windows to the outside and rooms that were not connected to the outside revealed a significant difference (p < 0.0001) (**Figure 6C**). In addition, a significant difference was found between rooms containing windows, rooms containing no windows and the anteroom (p < 0.0001). The anteroom, with >10 air changes/h, *A. fumigatus* CFUs were zero except for four samples (1, 1, 2, and 3 CFUs). For other fungi, no CFUs could be detected in the anteroom or patients rooms adjacent, except in three samples for *Penicillium* (1, 1, and 9 CFUs) (**Supplemental Data 1**).

## Discussion

Environmental air sampling was performed at the Manchester Adult Cystic Fibrosis Centre over a period of 14 months. Indoors and outdoor areas were sampled for different fungal species. *A. fumigatus* and *Penicillium* were the most dominant species in air samples, in line with previous studies (Shelton et al., 2002). *A. fumigatus* presence in the air correlated with days with elevated temperatures (sampling day and day before sampling) and with low wind speed. These data suggest *A. fumigatus* spores in the air are more abundant during the summer months, which is driven by increased temperatures and lower wind speeds. Indoor counts directly correlated to outdoor *A. fumigatus* counts and were elevated in rooms that directly connected to the outdoor *via* a window.

This study has demonstrated a positive correlation between ambient temperature and fungal presence, especially *A. fumigatus*, in environmental air samples. Other studies have found similar correlations (Li and Kendrick, 1995; Takahashi, 1997; Brenier-Pinchart et al., 2009; Alshareef and Robson, 2014) in different environmental settings. Adding further granularity to these observations, we draw positive correlations with the maximum and mean temperatures on the day of sampling, and also the day before sampling. Wind also appears to play a role in the spread of fungal spores (Grinn-Gofroń et al., 2018). Here we identified a negative correlation between wind speed and fungal CFUs. Previous studies have correlated effect of wind speed to fungal load (Takahashi, 1997; Brenier-Pinchart et al., 2009; Grinn-Gofroń et al., 2011), but no consistent pattern was found, potentially because the focus of fungal species differed between studies. Sequential events may give rise to increases in fungal counts in air samples. For *Coccidioides* spp., the “grow and blow” hypothesis has been put forward to facilitate fungal spread. Rainfall promotes growth within the soil, followed by a dry spell with elevated temperatures driving sporulation and aerosolization of spores (Comrie and Glueck, 2007; Tamerius and Comrie, 2011). Undergoing sporulation facilitates adaptation and evolution to environmental conditions (Zhang et al., 2015). Therefore, it is likely that elevated temperatures drive fungal sporulation leading to increased spores in the air. It is currently unclear what climatic factors affect different fungal species. However, temperature has been put forward as a universal driver of fungal proliferation (Alshareef and Robson, 2014; Chaloner et al., 2020). As climate change will drive more days with higher temperatures, this may have drastic effects on fungal loads in air samples (Stott, 2016).

Patients with CF are at risk of *Aspergillus* related pulmonary complications (Hong et al., 2018) but it is not known if there is a seasonal influence on incidence of ABPA or other *Aspergillus* related conditions in the CF population. Studies have reported seasonal influences on rates of pulmonary infections caused both by viruses and *Pseudomonas aeruginosa* in CF patients (Collaco et al., 2011; Flight et al., 2014). *P. aeruginosa* acquisition in young children with CF is more observed during summer months (Psoter et al., 2013). However, in a Danish retrospective study chronic infections were more common during winter months (Johansen and Høiby, 1992). *P. aeruginosa* and *A. fumigatus* can cause co-infection in CF and have been identified simultaneously in up to 60% of CF patients (Bakare et al., 2003; Paugam et al., 2010; Reece et al., 2017). In addition, increased air pollution is considered a risk factor for pulmonary exacerbations in cystic fibrosis patients (Goss et al., 2004). Elevated air pollution and temperature have a synergistic effect on each other (Ren et al., 2006). However, it is unclear how increased temperature has an effect on exacerbations caused by fungal spores.

Our data demonstrate that rooms with windows have significantly higher *A. fumigatus* counts compared to rooms without windows or with positive ventilation. It was unclear during our sampling; which windows were opened and the frequency of therefore should be monitored for future studies. Other interventions such as HEPA filters and laminar air flow systems in rooms have been proposed to keep the air free of fungi (Cornet et al., 1999; Hahn et al., 2002; Araujo and Cabral, 2010; Garnaud et al., 2012). At the MACFC, rooms with positive pressure (>10 ACHs) did not reach high levels of fungal spores (9 CFUs maximum). With the exception of four samples no fungal CFUs could be detected at all in these rooms. Increased presence of fungi in the air has been directly linked to the incidence of invasive aspergillosis in patients with other health conditions (Alberti et al., 2001; Brenier-Pinchart et al., 2011).

Our study consists of samples taken over 18 months. Longer sampling would allow assessing yearly seasonal variability and the relation with fungi in the air, which has not been studied in much detail (Calvo et al., 1980; Adhikari et al., 2004; Alshareef and Robson, 2014). We were able to detect and identify *A. fumigatus, Penicillium* species, *Geotrichum candidum*, and a mixed population of non-sporulating environmental filamentous fungi. Air samples were incubated at 30°C, which might bias towards fungi with their optimum growth at this temperature (Robert et al., 2015). Lastly, fungal CFUs were correlated to meteorological data collected from the weather station. However, we are unable to assess what parameters are directly causing increased fungal CFUs in the air. Further studies are required to find causative proof of climate and weather affecting fungal proliferation.

In summary, this study demonstrates that *A. fumigatus* spores in the air are more abundant during the summer months, which is driven by increased temperatures and lower wind speeds. Indoor counts directly correlated to outdoor *A. fumigatus* counts and were elevated in rooms that directly connected to the outdoor *via* a window. Further studies are required to determine the clinical implications of these findings for cystic fibrosis patients who are predisposed to *Aspergillus* related diseases, and in particular whether there is seasonal influence on incidence of *Aspergillus* related conditions and if screening for such complications such be increased during summer months and precautions intensified for those with a known history of *Aspergillus* related disease.

## Data Availability Statement

The original contributions presented in the study are included in the article/**Supplementary Material**. Further inquiries can be directed to the corresponding author.

## Author Contributions

NR, JC, LC, CM, MR, RB-T, and AJ contributed to conception and design of the study. LC and JC organized the database. NR performed the statistical analysis. NR wrote the first draft of the manuscript. NR, JC, LC, MD, RB-T, and AJ wrote sections of the manuscript. All authors contributed to the article and approved the submitted version.

## Funding

This research was funded by the Wellcome Trust, grant number 219551/Z/19/Z.

## Conflict of Interest

The authors declare that the research was conducted in the absence of any commercial or financial relationships that could be construed as a potential conflict of interest.

## Publisher’s Note

All claims expressed in this article are solely those of the authors and do not necessarily represent those of their affiliated organizations, or those of the publisher, the editors and the reviewers. Any product that may be evaluated in this article, or claim that may be made by its manufacturer, is not guaranteed or endorsed by the publisher.

## Supplementary Material

Supplementary Figure 1 Correlation of fungal CFUs on the sampling day and the day before sampling with all measured meteorological parameters. Spearman’s rank correlation of CFUs from fungi in outdoor air samples to meteorological parameters collected on the day of sampling or the day before sampling. Statistically significant (P < 0.05) correlations are shown in circles, with blue showing positive correlations and red negative correlations. Correlation values are shown and the size of the circle corresponds to this value.

Supplementary Figure 2 Correlation of fungal CFUs during the growing season with all measured meteorological parameters. Spearman’s rank correlation of CFUs from fungi in outdoor air samples to meteorological parameters during the growing season. Statistically significant (P < 0.05) correlations are shown in circles, with blue showing positive correlations and red negative correlations. Correlation values are shown and the size of the circle corresponds to this value.

Supplementary Figure 3
*A. fumigatus* CFUs are not associated with wind direction. *A. fumigatus* CFUs are shown for the wind direction during sampling.
