# Supplementary figures and images for: Meteorological Factors Influence the Presence of Fungi in the Air; A 14-Month Surveillance Study at an Adult Cystic Fibrosis Center
Source: Front Cell Infect Microbiol. 2021 Nov 26;11:759944. doi: 10.3389/fcimb.2021.759944 (PMC8662344; doi:10.3389/fcimb.2021.759944)

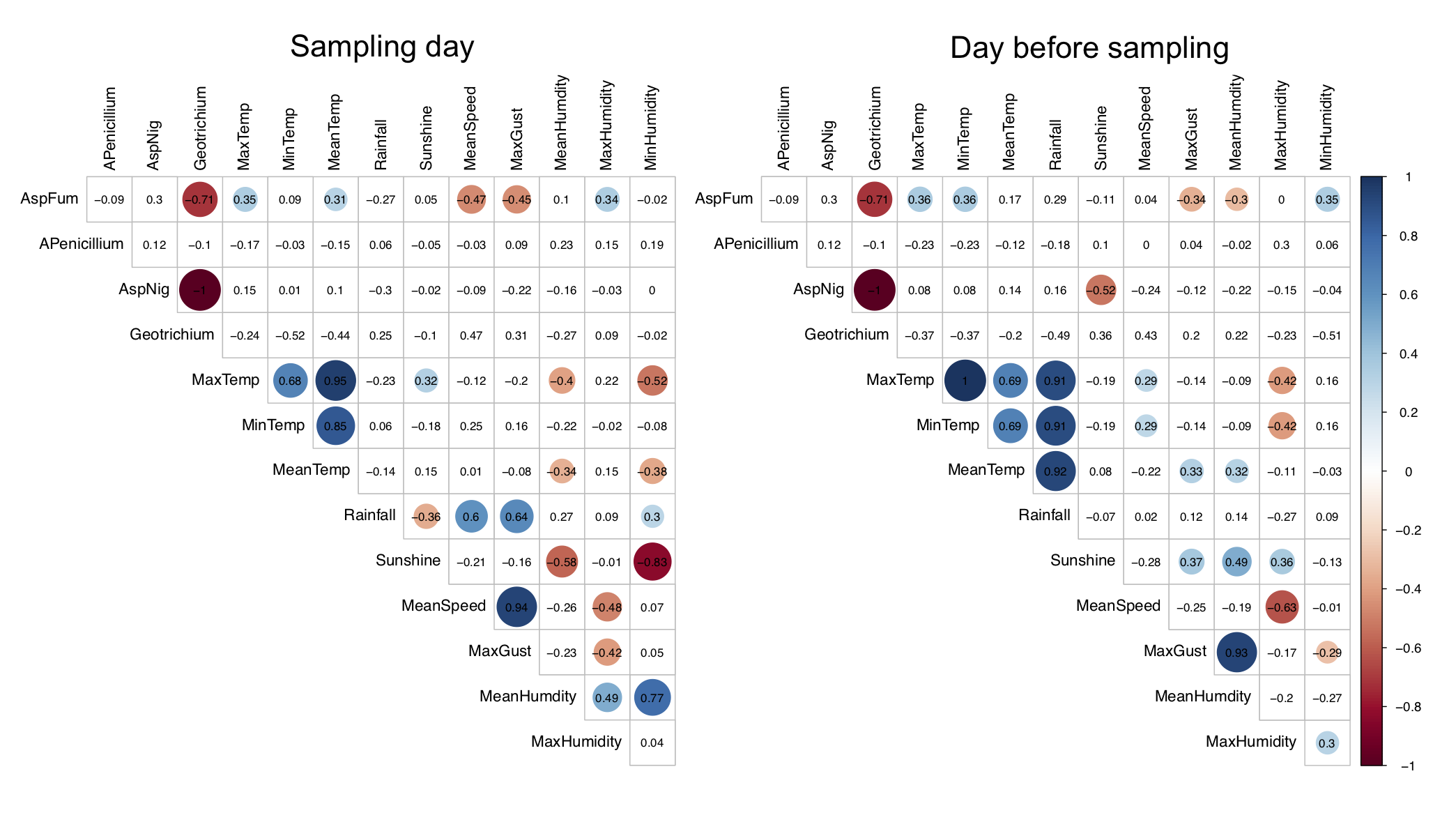

Supplement: Supplementary Figure 1 — Correlation of fungal CFUs on the sampling day and the day before sampling with all measured meteorological parameters. Spearman’s rank correlation of CFUs from fungi in outdoor air samples to meteorological parameters collected on the day of sampling or the day before sampling. Statistically significant (P < 0.05) correlations are shown in circles, with blue showing positive correlations and red negative correlations. Correlation values are shown and the size of the circle corresponds to this value. [file Image_1.tiff]

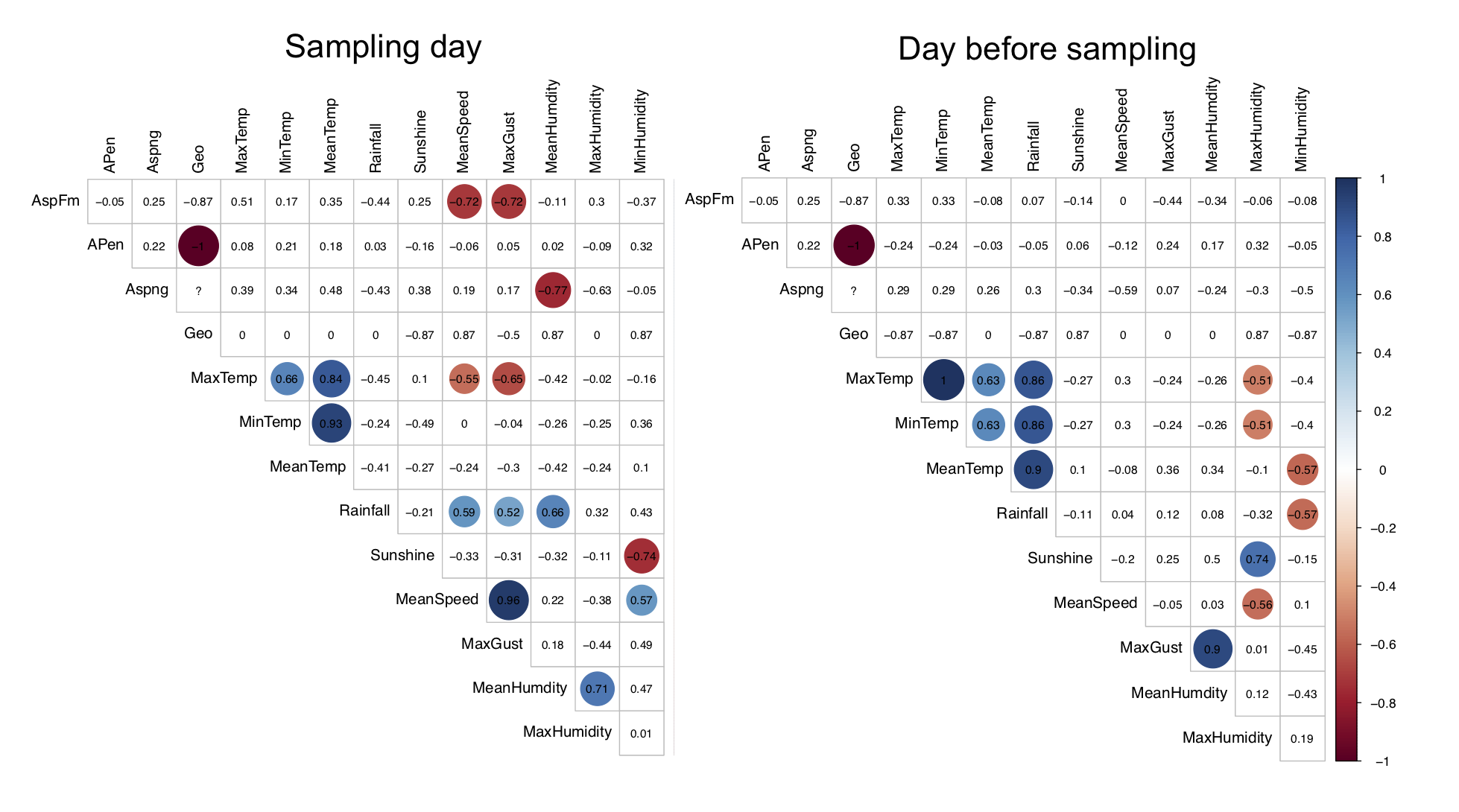

Supplement: Supplementary Figure 2 — Correlation of fungal CFUs during the growing season with all measured meteorological parameters. Spearman’s rank correlation of CFUs from fungi in outdoor air samples to meteorological parameters during the growing season. Statistically significant (P < 0.05) correlations are shown in circles, with blue showing positive correlations and red negative correlations. Correlation values are shown and the size of the circle corresponds to this value. [file Image_2.tiff]

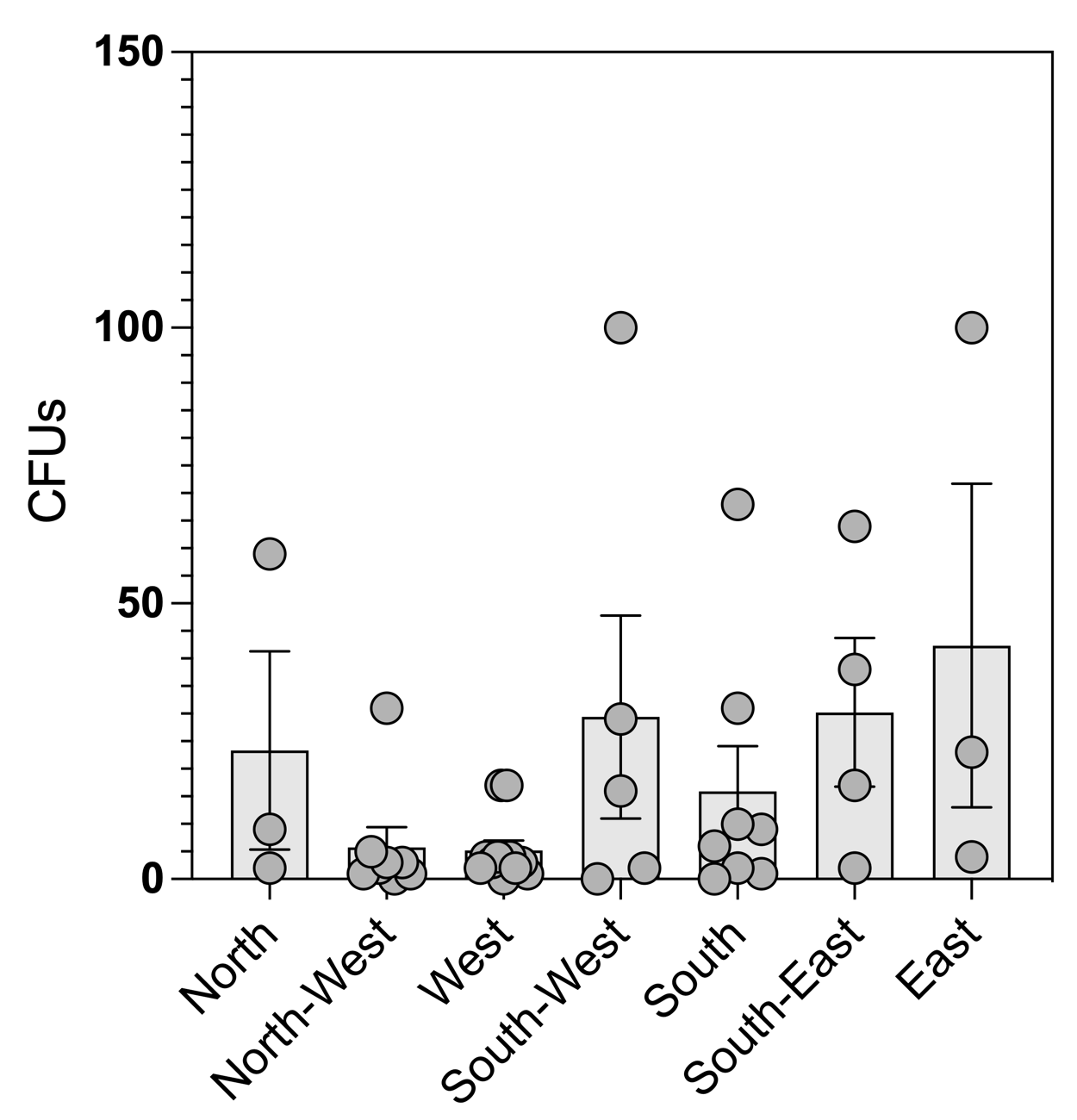

Supplement: Supplementary Figure 3 — A. fumigatus CFUs are not associated with wind direction. A. fumigatus CFUs are shown for the wind direction during sampling. [file Image_3.tiff]
